# Brainstem and Cerebellar Involvement in Ramsay Hunt Syndrome

**DOI:** 10.1155/2019/7605056

**Published:** 2019-12-12

**Authors:** Vijay Letchuman, Charles D. Donohoe

**Affiliations:** Department of Neurology, University of Missouri–Kansas City School of Medicine and Truman Medical Center, Kansas City, MO, USA

## Abstract

We present a case of a 62-year-old Caucasian male with laryngeal cancer and Ramsay Hunt Syndrome otherwise known as herpes zoster oticus due to reactivation of the varicella zoster virus. Classic findings include the triad of ipsilateral facial paralysis, otic pain, and herpetic lesions in the sensory supply of the facial nerve. The common pathogenesis is associated with anterograde axonal reactivation of the varicella zoster virus in the geniculate ganglion. Unique features of our case include retrograde transaxonal spread of the varicella-zoster virus from the geniculate ganglion into the brainstem and cerebellum including involvement of the abducens nucleus, facial nucleus, middle cerebral peduncle, and inferior cerebellar peduncle. This presented as left facial paralysis, left sixth nerve palsy, horizontal diplopia to the left lateral gaze, profound truncal ataxia, and left-sided dysmetria. Clinical awareness that Ramsay Hunt syndrome may also involve the brainstem and cerebellum is critical in evaluating the clinical neurologic findings and expanding the diagnostic workup to include brain magnetic resonance imaging and cerebrospinal fluid analysis, including varicella zoster polymerase chain reaction. Encephalitis requires longer duration administration of high-dose intravenous acyclovir in conjunction with steroids. Delays in treatment are often associated with unsatisfactory outcomes with extensive residual deficits.

## 1. Background

Varicella zoster virus (VZV) is a human herpes virus that, during the primary infection, causes chickenpox [1]. After the primary infection, the virus lies dormant in the ganglion of the sensory neurons. When reactivated, the virus produces herpes zoster, a painful, erythematous, vesicular rash in a dermatomal distribution. Common dermatomal locations involved are the thoracic (57%), cervical (20%), and trigeminal (15%) including the ophthalmic and lumbosacral (11%) [2]. VZV involvement of the geniculate ganglion is known as Ramsay Hunt syndrome (RHS), characterized by ipsilateral facial paralysis, otic pain, and herpetic lesions of the external auditory canal and auricle [3]. To the best of our knowledge, there have been 3 prior cases of RHS complicated exclusively by cerebellitis [1, 4, 5] and 3 prior cases of RHS complicated exclusively by rhombencephalitis [6–8]. While isolated involvement of the brainstem, cerebellum, and spinal cord has been discussed in the literature, no reports to our knowledge have described concurrent brainstem and cerebellar involvement [9].

## 2. Case Presentation

**Figure 1:**
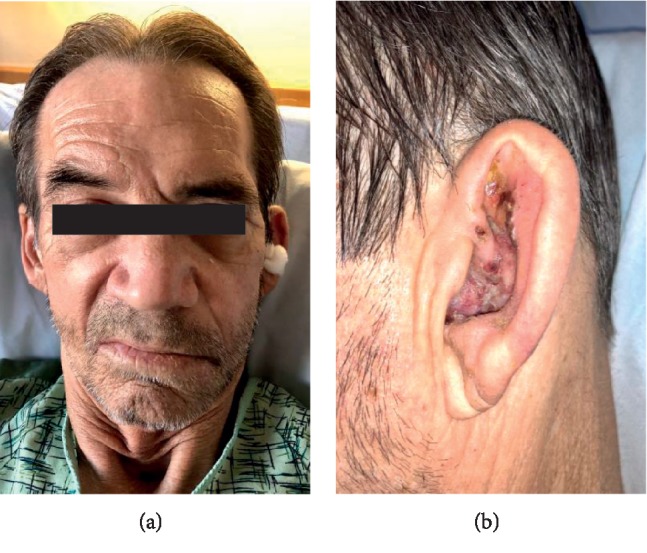
(a) Full left facial droop. (b) Left otic vesicles on the pinna.

A 62-year-old Caucasian male with a medical history significant for squamous cell laryngeal cancer in remission, history of tracheostomy, type 2 diabetes mellitus, and hypothyroidism presented to an outside institution with a 3-day history of left ear pain with associated nausea and vomiting. Initial laboratory studies demonstrated elevated nonspecific acute phase reactants. Computed tomography (CT) indicated left middle ear swelling and enhancement of the left soft palate. Fungal cultures were negative. A diagnosis of left malignant otitis externa was made, and appropriate intravenous and topical antimicrobial therapies were initiated. Due to clinical deterioration, the patient was transferred to our institution where initial neurological assessment showed a full left side facial droop, left otic vesicles on the pinna, and gait ataxia. Repeat CT scan was consistent with malignant otitis externa. On hospital day two, the patient's condition continued to worsen and neurological exam indicated left cranial nerve (CN) VI and VII nerve palsies, horizontal diplopia of the left eye, conductive hearing loss, skew deviation, and persistent herpetic lesions on the hard palate and the outer ear (Figure 1). There was no nystagmus, and clinical head impulse exam was negative.

**Figure 2:**
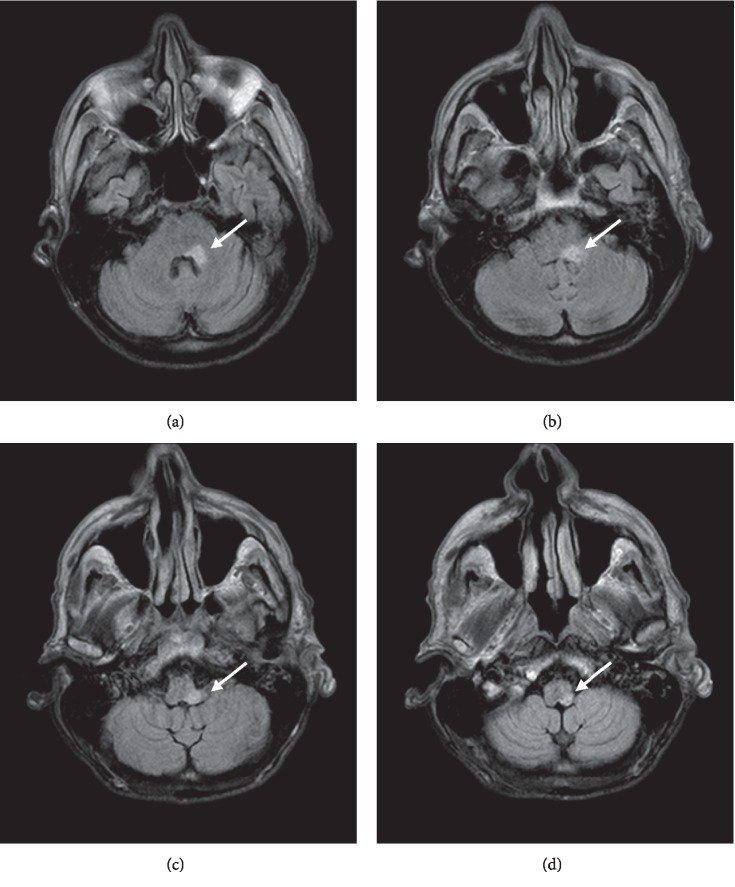
FLAIR (fluid-attenuated inversion recovery) images. (a) Focus of increased signal intensity noted at the level of the fourth ventricle involving the left CN VI (abducens) nucleus, left CN VII (acial) nucleus, and left middle cerebellar peduncle. (b) Focus of increased signal extends caudally to the level of the cerebellar vermis involving the middle cerebellar peduncle. (c) Further to the level of the cerebellar vermis with involvement of the medulla oblongata and left pyramid. (d) Terminating at the level of the cerebellar tonsils involving the left inferior cerebellar peduncle (restiform body).

**Figure 3:**
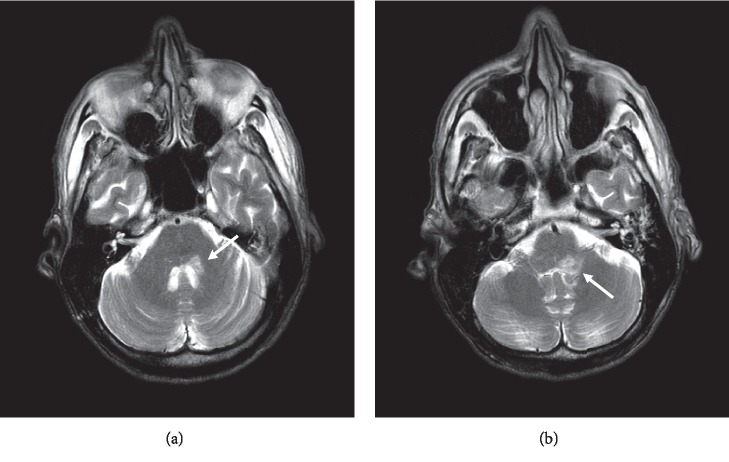
T2 images. (a) Focus of the increased signal at the level of the fourth ventricle involving the middle cerebellar peduncle. (b) Focus of the increased signal at the level of the cerebellar vermis involving the inferior cerebellar peduncle.

**Figure 4:**
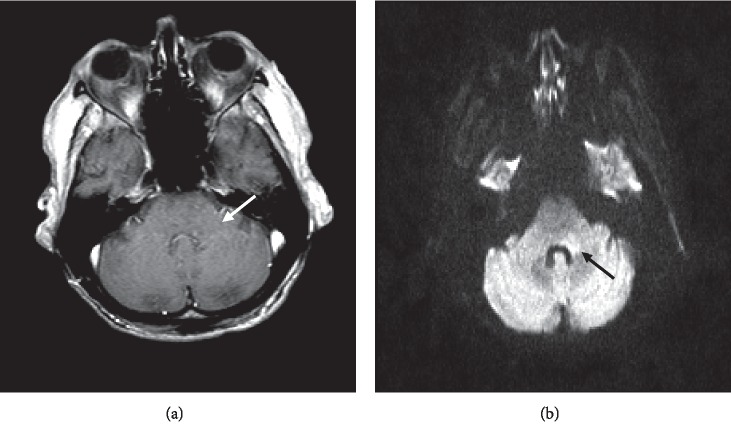
(a) T1 postcontrast image: lesion demonstrates lack of contrast enhancement. (b) Diffusion-weighted image (DWI) b1000: lesion demonstrates area of restricted diffusion at the level of the fourth ventricle involving the middle cerebellar peduncle.

Due to suspected multiple CN involvement, contrast brain magnetic resonance imaging (MRI) was conducted followed by a lumbar puncture (LP). Brain MRI showed a hyperintense lesion along the left middle and inferior cerebellar peduncle along the fourth ventricle (Figures 2–4). Cerebrospinal fluid (CSF) revealed elevation in glucose, protein, and nucleated cells (lymphocyte-predominant). VZV quantitative polymerase chain reaction (PCR) tested positive. Herpes simplex virus (HSV) 1 and 2 titers tested negative (Table 1). Diagnosis of Ramsay Hunt syndrome (RHS) with concomitant VZV encephalitis was made at this time.

Initial treatment included empiric antibiotic and antifungal medications; however, after the confirmation of viral etiology, these were discontinued. Intravenous acyclovir 800 mg 5x/day and prednisone 70 mg oral daily was initiated. One day later, the patient showed significant improvement in visual deficits. Mild lateral gaze palsy remained along with full left facial weakness, gait ataxia, and vesicular lesions on the hard palate and ear. He remained in the hospital for 9 days and was discharged on acyclovir 20 mL 5x/day for 14 days.

At 4-month follow-up, the patient demonstrated improved balance but had persistent left facial droop and left-sided hearing loss. The remainder of the neurological exam was unremarkable. Due to the recurrence of left otic discharge, the patient was discharged on a 10-day course of 1 g valacyclovir three times/day as an outpatient.

## 3. Discussion

We describe a patient with both RHS along with a complicating VZV rhombencephalitis and cerebellitis. Prior to making the diagnosis of RHS, there was concern that the cause of the patient's presentation could be secondary to direct metastasis of his squamous cell laryngeal carcinoma. The potential for malignancy was vaguely supported by the nonspecific contrast enhancement on brain imaging; however, it was excluded after the CSF evaluation was completed. Also the differential diagnosis at the initial stages was direct neurotoxicity secondary to the patient's cisplatin chemotherapy regimen. Considering the classic presentation associated with Ramsay Hunt syndrome, cerebellar involvement, and the lack of distal sensory neuropathy, this was excluded as a potential diagnosis [10].

Central nervous system (CNS) involvement is unusual in Ramsay Hunt syndrome but is associated with immunocompromised states [11]. Clinical signs, imaging, and CSF studies confirmed CNS involvement in our patient. T-cell-mediated immunity is required for maintaining the latency of VZV in the sensory ganglion [12]. Immunocompromised patients due to immunosuppressant agents, chemotherapy, or other etiologies have an increased risk of disseminated VZV infection including encephalitis [13]. Immunosuppressants impair leukocyte adhesion and migration into the CNS, potentially suppressing the natural defense against VZV encephalitis [1]. The patient, in this case, had recently completed a course of chemotherapy for squamous cell laryngeal cancer (4 months prior to presentation) which likely led to an immunocompromised state, predisposing him to the development of VZV reactivation. Clinical signs, imaging, and CSF studies confirmed CNS involvement in our patient. Our patient presented with signs indicative of both cerebellar and brainstem involvement considering the multiple cranial nerve signs. Cerebellar peduncle involvement in this case was likely the cause of the patient's significant gait ataxia. While imaging came back conclusive for a cerebellar lesion in our patient, if negative in another case, imaging cannot be dismissed since the sensitivity of MRI in detecting VZV lesions is largely unknown, due to the limited data [14]. The route of infection to the CNS is unclear, but research postulates spreading through hematogenous or CSF pathways [5].

Diagnosis in the cases of concomitant RHS and VZV encephalitis generally consists of the clinical features, brain MRI, and CSF analysis, all of which were used in this case to establish a diagnosis [1, 15]. However, the sensitivity of these tests has not been well established in the literature. In our case, all three diagnostic modalities were necessary due to the unusual nature of the presentation. One of the peculiar points of our case in comparison with most cases of VZV encephalitis was that in our case, there were no features of vasculitis. The typical features of VZV encephalitis often present as an ischemic infarction with arterial stenosis [14]. In our patient, MRI did not support the presence of vasculitis and therefore an angiogram was not performed for our patient. Notably, in patients that do undergo an angiogram, a negative result does not rule out VZV vasculitis since angiogram is not sensitive to small-vessel vasculitis [14].

The standard treatment of RHS itself focuses on acyclovir and prednisone therapy. Of utmost importance in these cases is the timeframe during which the infection is treated. In the study performed by Furuta et al., it was found that RHS patients who received treatment with the combination acyclovir-prednisone within 7 days of clinical presentation had a full facial palsy recovery [16]. Murakami et al. found that patients treated with the acyclovir-prednisone combination within 3 days of onset had a 75% recovery rate versus the 30% recovery rate in patients where therapy was initiated more than 7 days after initial onset [17]. The timeframe of treatment is the key point of discussion in our case due to the delay in initiation of acyclovir-prednisone therapy. As a result of the unusual presentation of the patient's symptoms and the delay in the onset of classic symptoms indicative of RHS, combination therapy was not initiated until 7 days after initial presentation. Prognosis is controversial but seems to be related to the time span between onset and initiation of therapy. Facial paralysis recovery rates vary between studies but range from 10 to 75% recovery rate [1, 16, 17]. The wide variation in prognosis is likely due to the speed of treatment along with clinical status at the time of presentation. Immunocompromised patients are likely to be affected by more severe infections and therefore likely to have persistent residual deficits. The patient in our case had a recurrence of otic discharge at his 4-month follow-up, which may have been secondary to the immunosuppressive effects of the prednisone therapy [1].

## Figures and Tables

**Table 1:** Cerebrospinal fluid (CSF) results.

Spinal fluid	Value	Reference range
Appearance	Clear	
Glucose	120 mg/dL	40–70 mg/dL
Protein	75 mg/dL	15–45 mg/dL
Lymphocytes	81%	—
Neutrophils	10%	—
Monocytes	9%	—
Serology	Result	Reference range
Varicella zoster virus PCR^*∗*^	465,717	<500
Herpes simplex virus 1 IgM^*∗∗*^	Nonreactive	—
Herpes simplex virus 2 IgM^*∗∗*^	Nonreactive	—

^*∗*^PCR: polymerase chain reaction; ^*∗∗*^IgM: immunoglobulin.

## Abbreviations

CN: Cranial nerve
CNS: Central nervous system
CSF: Cerebrospinal fluid
CT: Computerized tomography
DWI: Diffusion-weighted image
FLAIR: Fluid-attenuated inversion recovery
HSV: Herpes simplex virus
MRI: Magnetic resonance imaging
PCR: Polymerase chain reaction
RHS: Ramsay Hunt syndrome
VZV: Varicella zoster virus.
